# Infrared Spectrum of the *v*_2_+ *v*_6_ Band of C^13^C^12^H_6_*

**DOI:** 10.6028/jres.067A.021

**Published:** 1963-06-01

**Authors:** Walter J. Lafferty, Earle K. Plyler

## Abstract

The infrared spectrum of the *v*_2_*+v*_6_ band of C^13^C^12^H_6_ has been analyzed and a value of *B*_0_= 0.64865 ±0.00005 cm^−1^ determined. When this value is combined with that found in recent work on isotopically normal ethane, a *“r_s_”* value of 1.527±0.004 A for the carboncarbon bond distance is obtained. (Uncertainties are probable errors.)

## 1. Introduction

From recent infrared studies of ethane and ethane-*d*_6_, a ground state carbon-carbon bond distance of 1.536 with probable error 0.002 A has been obtained [1, 2].^1^ This bond distance is considerably higher than the C—C bond lengths, 1.526 with limits of error ±0.002 A, obtained for the saturated hydrocarbons propane and isobutane by the “*r_s_”* or “substitution” method in the microwave studies of Lide [3, 4]. Since the two methods of determining bond distances are only approximately equivalent due to rotation-vibration effects, this difference is not surprising.

The purpose of this work was to examine the spectrum of C^13^C^12^H_6_ in an attempt to obtain a “*r_s_*” value for the C—C bond distance of ethane for comparison with these bond lengths reported for the more complex molecules. In addition other rotational constants would be obtained which would be of use to future investigators of this molecule.

## 2. Experimental Procedure

The sample, which contained 59.5 percent C^13^C^12^H_6_, was purchased from Merck Sharp & Dohme of Canada, Ltd. The spectra were taken with a pressure of 3.2 mm Hg total pressure (1.9 mm C^13^C^12^H_6_) and an optical path of 24 m.

The spectrometer, used in this study as well as for the normal ethane work [2], has been described previously. Because of the small amount of costly sample available, a threefold longer optical path (24 m) was required than was used for ethane. This resulted in the loss of some resolution; however, lines separated by about 0.035 cm^−1^ could be resolved. The spectra were measured by using rare gas emission lines as standards. The regions between the standard lines were measured from the fringe system formed by a Fabry-Perot interferometer [5].

## 3. Rotational Analysis

As in the case of normal ethane, the only parallel band of C^13^C^12^H_6_ that could be resolved sufficiently well with the instrumentation available was the *v*_2_+*v*_6_ band at 2749 cm^−1^. Substitution of one C^13^ in ethane results in a shift of about 4.1 cm^−1^ for the origin of this band. Since the sample contained about 40 percent normal ethane, the resulting spectrum consisted of a complicated mixture of lines from the two overlapping bands together with their accompanying “hot bands” arising from excited levels of the torsional vibration. The lines of ethane were easily identified when the spectra were compared with those obtained from the earlier studies on ethane. The vibrational shift was such that most of the *P* and *R* branch lines fell between those of the normal ethane. There was, however, some blending of the lines. Since blending can result in an apparent change of the frequency of a line, all blended lines were marked as such when assigned and given a weight of ½ in the following calculations. Badly overlapped lines were not used in the analysis.

As in the case of normal ethane, the quantity (*A′ – B′*) – (*A″–B″*) was sufficiently large so that transitions from the substates *K=2* and higher were resolved. The unresolved lines from the substates *K*=0 and 1 were not used in the calculations. The ground state rotational constants were calculated by means of combination differences from the equation

$$\begin{array}{l} \Delta_{2}F^{\prime \prime }=R(J-1,K)-P(J+1,K)\\ \;=(4B^{\prime \prime }-6D_{J}^{\prime \prime }-4D_{JK}^{\prime \prime }K^{2})(J+\frac{1}{2})\\ \;-8D_{J}^{\prime \prime }{\left(J+\frac{1}{2}\right)}^{3}. \end{array}$$ (1)

Since no substates with *K* greater than 6 were identified, the value of $D_{JK}^{\prime \prime }$ obtained was highly uncertain. The assumption was then made that this constant is equal to that found for ethane times the ratio of the *B* values of the substituted ethane to that of the normal molecule or 5.8×10^−6^ cm^−1^. With this assumed constant, the resulting ground state rotational constants found by least squares were *B*″:=0.64865±0.00005 cm^−1^ and $D_{J}^{\prime \prime }=8.4\pm 1.4\times 10^{-7}$ cm^−1^ where the uncertainties cited are probable errors. The values of $D_{J}^{\prime \prime }$ calculated is in good agreement with that found for normal ethane ( $D_{J}^{\prime \prime }=7\pm 2\times 10^{-7}$ cm^−1^.

The upper state rotational constants were determined using the equation

$$\begin{array}{l} v=v_{0}+[B^{\prime }+B^{\prime \prime }-(D_{JK}^{\prime }+D_{JK}^{\prime \prime })K^{2}]m+[B^{\prime }-B^{\prime \prime }\\ \;-D_{J}^{\prime }+D_{J}^{\prime \prime }-(D_{JK}^{\prime }-D_{JK}^{\prime \prime })K^{2}]m^{2}-2(D_{J}^{\prime }+D_{J}^{\prime \prime })m^{3}\\ \;-(D_{J}^{\prime }-D_{J}^{\prime \prime })m^{4}+[(A^{\prime }-B^{\prime })-(A^{\prime \prime }-B^{\prime \prime })]K^{2}. \end{array}$$ (2)

The values of *B*″, $D_{J}^{\prime \prime }$, $D_{JK}^{\prime \prime }$ obtained above were inserted into the equation, and the data were subjected to a least squares fit. As in the case of normal ethane, the *K*=5 substate was found to be perturbed. The origin of this state fell 0.03_2_ cm^−1^ above the calculated value. While this difference is small, it is 3.1 times the standard deviation of the fit taken without including this substate and is therefore significant. Any changes in the other rotational constants of this state were too small to be detected. Because of this perturbation, the observed frequencies were again fit to eq (2) with the frequencies from the *K*=5 substate omitted. The constants obtained from this treatment are listed in table 2. The spectrum calculated from these constants is compared with the observed spectrum in table 1. For the *K*=5 substate, the observed origin was used instead of that calculated from eq (2).

## 4. Results

For a symmetric top molecule, the “*r_s_*” distance of an atom on the symmetry axis from the center of gravity is given by the relation

$$\Delta I_{0}=\mu r_{s}^{2}$$ (3)

where Δ*I*_0_ is the change of the moment of inertia upon substitution, *μ=m*Δ*m*/(*M*+2*m*), *M* is the mass of the original molecule, and Δ*m* is the change of mass upon substitution. Using the *B*_0_ value obtained earlier for ethane and the value found for the C^13^ substituted ethane above, a “*r_s_*” value of 1.527± 0.004 A for the C—C distance is calculated. The uncertainty cited is probable error.

This value supports hide’s estimate that the *“r_s_”* value for ethane lies in the range 1.525–1.530 A [6] and is in good, albeit somewhat fortuitous, agreement with the value 1.526 A found for the C—C bond lengths in propane and isobutane.

## Figures and Tables

**Table 1:** Comparison between observed and calculated spectrum of v_2_+v_6_ of *C*^13^*C*^12^*H*_6_

*J_K_*	*^Q^R_K_*(*J*)		*^Q^P_K_*(*J*)	
	Obs	Calc	Obs	Calc
				
	*cm*^−1^	*cm*^−1^	*cm*^−1^	*cm*^−1^
3_0_	}2754.164	$\left\{ \begin{array}{l} 2754.141\\ 54.163 \end{array}\right.$	………	………
3_1_			………	………
3_2_	54.229	54.227	2745.290	2745.297
3_3_	b54.346	b54.336	………	………
4_0_	}54.362	$\left\{ \begin{array}{l} 55.331\\ 55.353 \end{array}\right.$	………	………
4_1_			………	………
4_2_	55.410	55.417	b43.931	43.936
4_3_	55.545	55.525	44.058	44.046
4_4_	55.671	55.675	………	………
5_0_	}56.528	$\left\{ \begin{array}{l} 56.501\\ 56.522 \end{array}\right.$	………	………
5_1_			………	………
5_2_	56.589	56.585	42.550	42.553
5_3_	56.719	56.69l	42.652	42.662
5_4_	56.838	56.840	42.825	42.814
5_5_	b57.072	57.061	………	………
6_0_	}57.671	$\left\{ \begin{array}{l} 57.650\\ 57.669 \end{array}\right.$	………	………
6_1_			………	………
6_2_	57.728	57.732	41.158	41.149
6_3_	57.860	57.836	41.276	41.257
6_4_	57.977	57.982	b41.411	41.407
6_5_	58.195	………	41.646	41.635
6_6_	58.399	58.399	………	………
7_0_	}2758.800	$\left\{ \begin{array}{l} 2758.775\\ 58.795 \end{array}\right.$	2739.661	$\left\{ \begin{array}{l} 2739.638\\ 39.657 \end{array}\right.$
7_1_				
7_2_	58.839	58.857	b39.722	39.724
7_3_	b58.963	58.959	39.835	39.830
7_4_	59.085	59.102	39.980	39.978
7_5_	59.342	59.317	40.199	40.203
7_6_	59.501	59.511	………	………
8_0_	}59.898	$\left\{ \begin{array}{l} 59.882\\ 59.901 \end{array}\right.$	38.209	$\left\{ \begin{array}{l} 38.193\\ 38.213 \end{array}\right.$
8_1_				
8_2_	59.950	59.961	38.205	38.277
8_3_	60.087	60.061	38.388	38.381
8_4_	60.191	60.201	38.537	38.527
8_5_	60.396	60.412	38.751	38.749
8_6_	60.589	60.600	38.944	38.944
9_0_	}60.988	$\left\{ \begin{array}{l} 60.966\\ 60.984 \end{array}\right.$	………	………
9_1_			………	………
9_2_	61.024	61.043	36.794	36.809
9_3_	61.152	61.140	36.913	36.912
9_4_	b61.277	61.276	37.055	37.055
9_5_	61.476	61.483	………	………
9_6_	61.664	61.666	37.468	37.463
10_0_	}2762.045	$\left\{ \begin{array}{l} 2762.037\\ 62.045 \end{array}\right.$	2735.269	$\left\{ \begin{array}{l} 2735.238\\ 35.258 \end{array}\right.$
10_1_				
10_2_	62.079	62.103	35.315	35.320
10_3_	62.203	62.197	35.433	35.420
10_4_	62.322	62.330	35.560	35.560
10_5_	62.534	62.531	35.757	35.773
10_6_	62.721	62.708	35.948	35.959
11_0_	}63.082	$\left\{ \begin{array}{l} 63.069\\ 63.086 \end{array}\right.$	33.756	$\left\{ \begin{array}{l} 33.731\\ 33.749 \end{array}\right.$
11_1_				
11_2_	63.150	63.141	33.802	33.810
11_3_	63.236	63.233	b33.908	33.907
11_4_	b63.356	63.361	34.044	34.043
11_5_	63.540	63.556	34.241	34.253
11_6_	63.717	63.728	34.440	34.433
12_0_	}64.104	$\left\{ \begin{array}{l} 64.089\\ 64.105 \end{array}\right.$	32.230	$\left\{ \begin{array}{l} 32.201\\ 32.218 \end{array}\right.$
12_1_				
12_2_	64.143	64.158	32.270	32.278
12_3_	b64.253	64.247	b32.369	32.373
12_4_	b64.368	64.370	32.509	32.505
12_5_	64.558	64.560	32.696	32.710
12_6_				
	64.715	64.723	32.890	32.883
13_0_	}65.111	$\left\{ \begin{array}{l} 65.087\\ 65.103 \end{array}\right.$	30.679	$\left\{ \begin{array}{l} 30.649\\ 30.667 \end{array}\right.$
13_1_				
13_2_	65.150	65.154	30.719	30.725
13_3_	b65.231	65.238	30.834	30.816
13_4_	b65.358	65.357	30.937	30.945
13_5_	65.529	65.540	31.150	31.144
13_6_	65.700	65.696	31.310	31.311
14_0_	}………	………	2729.112	$\left\{ \begin{array}{l} 2729.007\\ 29.095 \end{array}\right.$
14_1_				
14_2_	………	………	29.142	29.151
14_3_	2766.237	2766.208	29.239	29.239
14_4_	b66.310	66.321	29.363	29.363
14_5_	66.508	66.497	29.542	29.556
14_6_	66.638	66.645	………	………
				
15_2_	67.070	67.079	27.548	27.556
15_3_	………	………	b27.618	27.641
15_4_	b67.259	67.263	27.755	27.759
15_5_	67.444	67.431	27.952	27.947
15_6_	67.591	67.571	28.097	28.098
				
16_0_	}67.976	$\left\{ \begin{array}{l} 67.952\\ 67.964 \end{array}\right.$	25.894	$\left\{ \begin{array}{l} 25.872\\ 25.888 \end{array}\right.$
16_1_				
16_2_	68.010	68.009	25.935	25.940
16_3_	………	………	26.030	26.021
16_4_	………	………	26.137	26.134
16_5_	68.330	68.342	………	………
16_6_	68.468	68.473	………	………
				$\left\{ \begin{array}{l} 24.238\\ 24.252 \end{array}\right.$
17_0_	}………	………	24.268	
17_1_				
17_2_	………	………	24.298	24.302
17_3_	68.990	68.985	24.385	24.379
17_4_	………	………	24.487	24.487
17_5_	69.236	69.234	………	………
17_6_	69.356	69.352	………	………
18_2_	………	………	22.649	22.644
18_3_	………	………	22.707	22.716
18_4_	………	………	22.818	22.818
19_3_	………	………	2721.030	2721.032
19_4_	………	………	21.129	21.127
20_2_	………	………	19.270	19.264
20_3_	………	………	19.348	19.327

b Blended line.

**Table 2:** Rotational constants of *C*^13^*C*^12^*H*_6_ derived from the combination band, v_2_ + v_6_ in *cm*^−1^^a^

*v*_0_	2749.164± 0.002
$B^{\prime \prime }$	0.64865±0.00005
$D_{J}^{\prime \prime }$	8.4±1.4×10^−7^
$D_{JK}^{\prime \prime }$	5.8×10^−6^ (assumed)
$B^{\prime }-B^{\prime \prime }$	−0.01062±0.00001
$D_{JK}^{\prime }-D_{JK}^{\prime \prime }$	3.12± 0.07×10^−5^
$D_{J}^{\prime }-D_{J}^{\prime \prime }$	~0
$D_{K}^{\prime }-D_{K}^{\prime \prime }$	~0

(*A*′–*B*′)–(*A*″–*B*″) = 0.0226±0.0005	

a Uncertainties cited are probable errors.

## Footnotes

1 Figures in brackets indicate the literature references at the end of this paper.
